# Ergonomic Risk Assessment during an Informal Hand-Made Cookware Operation: Extending an Existing Model

**DOI:** 10.3390/ijerph18189459

**Published:** 2021-09-08

**Authors:** Busisiwe Shezi, Renee A. Street, Angela Mathee, Nokulunga Cele, Sipho Ndabandaba, Rajen N. Naidoo

**Affiliations:** 1 Environment and Health Research Unit, South African Medical Research Council, Johannesburg 2094, South Africa; angie.mathee@mrc.ac.za; 2 Department of Environmental Health, University of Johannesburg, Johannesburg 2094, South Africa; 3 Environment and Health Research Unit, South African Medical Research Council, Durban 4000, South Africa; renee.street@mrc.ac.za (R.A.S.); nokulungacele6@gmail.com (N.C.); ndabandabasp@gmail.com (S.N.); 4 Discipline of Occupational and Environmental Health, University of KwaZulu-Natal, Durban 4000, South Africa; naidoon@ukzn.ac.za; 5 Department of Environmental Health, Nelson Mandela University, Gqeberha 6000, South Africa

**Keywords:** hand-made cookware operation, ergonomics, risk assessment, informal work, action priority matrix

## Abstract

The work conducted in the informal sector is highly variable within and between days. Characterizing ergonomic exposures remains a challenge because of unstructured work settings and schedules. The existing ergonomic risk assessment tools have been widely used in formal work settings with a narrow range of exposure, and for predefined tasks that primarily constitute a daily routine. There is limited information in the literature on how they have been applied in informal workplaces. The aim of this study was to extend an existing risk assessment tool and to evaluate the applicability of the extended tool by assessing ergonomic exposure related to hand-made cookware operations. Eighteen hand-made cookware makers were recruited from six sites. A walkthrough risk assessment questionnaire was used to collect information on workers, tasks, work stations and workplace structures. The Rapid Upper Limb Assessment (RULA) screening tool was extended by including duration and vibration. An action priority matrix was used to guide intervention. According to the RULA action levels, the workers required investigation and changes soon, and immediate investigation and changes. The use of an action priority matrix was appropriate, and indicated that all the workers assessed were within the high to very high exposure domain and required immediate corrective measures. The methodology used proved to be an effective and reliable strategy for identifying ergonomic exposure among hand-made cookware makers.

## 1. Introduction

Epidemiological studies have reported a causal relationship between physical exertion at work (i.e., awkward postures, prolonged static work, repetitive movements, manual material handling, forceful exertions and vibration) [1,2,3] and work-related musculoskeletal disorders (WRMDs) [4]. The leading causes of years lived with disability have been reported for WRMDs [5], with low back pain being ranked as sixth in terms of disability-adjusted life-years (DALYs) [6]. Psychosocial factors such as stress, job dissatisfaction and time pressure have also been reported to be associated with WRMDs [7,8].

Various ergonomic risk assessment tools have been used to assess the tasks, postures, movement frequency, force and muscle use in the workplace. These include the Quick Exposure Check (QEC) [7], Rapid Entire Body Assessment (REBA) [9], Rapid Upper Limb Assessment (RULA) [10], Strain Index [11], Revised NIOSH lifting equation [12] and Ovako Working Posture Analysing System (OWAS) [13] (and the associated software WinOWAS) [14]. Recently developed exposure assessment tools include the Occupational Repetitive Actions (OCRA) [15], Upper Limb Risk Assessment (ULRA) [15,16,17] and Postural Ergonomic Risk Assessment (PERA) [18]. These tools have been used in various work settings to assess ergonomic risk factors related to musculoskeletal disorders. There is limited information in the literature on how they have been applied in informal workplaces. In particular, the most feasible way for analysing work postures has not been adequately assessed among informal workers. Nevertheless, studies conducted among informal workers have reported WRMDs related to the work profile and often times hazardous nature of the work. For example, in Thailand, 87% of the informal workers making handcrafts reported upper back pain [19]. In Brazil, 42% of the informal mine workers reported back pain [20]; in Ghana, e-waste collectors reported 90% overall WRMDs such as lower back pain (65%), knees (39%) and shoulders (37%) [21].

Several public and private occupational health interventions have been implemented in the formal sector; however, the informal sector continues to operate without defined control measures [22,23,24]. In addition, informal workers usually lack policy regulations and strict adherence to labor regulations and associated government regulations [22]. There are different types of risk assessment methodologies with varying levels of precision in accounting for occupational exposure variations; these broadly include self-reports, a subjective measure of workplace exposure based on the workers observations [25]. Direct measurement is a more precise measure of exposure and may provide real-time measures of exposure [26]. However, this method is more effective in controlled settings with predefined tasks and daily routines.

Observational methods are more widely used, cost-effective, and largely rely on the expertise of the observer, and may provide a critical insight into workplace exposure variations and thus an appropriate approach for informal settings [10]. An observational workplace assessment [27] conducted in Durban, South Africa reported prevalent ergonomic hazards among informal traditional medicine traders [27]; however, because of the variety of tasks with varying frequencies, duration and tools; ergonomic risk levels were not assessed. The development of risk assessment tools tailored for informal workers, which account for variation in exposure, were recommended in this study.

Hand-made cookware operation involves preparing sand molds for casting, and a smelting process to cast liquid aluminium melted from a collection of scrap metal [28,29,30]. Studies conducted among hand-made cookware makers have reported exposure to high levels of metals [30], and particulate matter [31]. However, ergonomic risks have not been assessed among these workers. The existing risk assessment tools have been widely used in formal work settings with a narrow range of exposure. Therefore, it is necessary to develop or adapt simple, cheap and easy to use ergonomic risk assessment tools for small scale self-employment operations such as hand-made cookware operations; that will make provisions for varying work schedules, tasks, duration and product demand. The aim of this study was to extend an existing risk assessment tool [10] and to evaluate the applicability of the extended tool by assessing ergonomic risk factors related to hand-made cookware operations.

## 2. Materials and Methods

This study was undertaken during June and July 2019. The target population was hand-made cookware makers situated in the provinces of Limpopo (Giyani) and Kwa-Zulu Natal (Durban), South Africa. Giyani is a city in the north-eastern part of the Limpopo province, while Durban is situated along the east coast of South Africa. A convenient date and time suitable for data collection were set with the hand-made cookware makers. At the appointment date, written consent was obtained from the workers willing to participate, at each site, before data were collected.

### 2.1. Data Collection

A questionnaire was administered face-to-face to collect information on demographic characteristics. A risk assessment observational questionnaire was used to assess the workers, workstations, workplace structure, risk factors and worker exposure to those risk factors. The workers had different workstations for performing their tasks, and used a range of postures (i.e., standing, bent over, sitting or kneeling). The selection of posture for each task was based on the postures sustained for the longest period (i.e., squatting when removing defects). To gain an understanding of the job tasks and demands, the workers’ movements were observed over repeated observations from the 24th of June 2019 to the 11th of July 2019. The workers were observed for a period of 8–10 h per day. Depending on the number of workers, size of the cookware and demand, the workers made two to 15 cookware products per working day. To assess muscle load, the weight of the objects was measured with digital weighing scales. The data collectors (observers) were trained by an occupational health and safety specialist prior to data collection. The observers and the occupational health and safety specialist undertook each session independently and the results were compared and consolidated. Where there was a difference in opinion, consensus was reached through discussion.

### 2.2. Data Analysis

Descriptive statistics such as means, standard deviation (SD), median (range) and percentages were calculated using Stata IC version 14 (StataCorp, College Station, TX, USA).

#### Rapid Upper Limb Assessment (RULA) Tool

(i) RULA—An Existing Ergonomic Risk Assessment Tool

**Figure 1:**
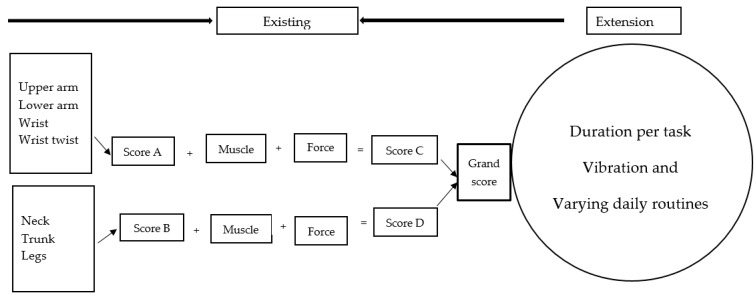
The RULA scoring sheet [10] extended to include duration, vibration and varying daily routines.

The Rapid Upper Limb Assessment (RULA) tool [10] was applied (Figure 1) using information collected during the walk-through observation. RULA is a screening tool designed to evaluate the load on the musculoskeletal system and it was developed to make an initial recommendation for a detailed assessment by evaluating the workers’ exposure to postures, forces and muscle activities. It is an upper limb tool, but it also investigates awkward or constrained postures of the legs to ensure that the whole body is assessed. Risk is quantified by (i) recording working postures; (ii) grouping the body part postures (a) upper arm, lower arm, wrist, wrist twist and additional load (force and muscle), (b) neck, trunk and legs and additional load (force and muscle); (iii) calculating the grand score; and (iv) evaluating an overall musculoskeletal grade to guide intervention (this is summarised into four RULA action levels (RALs), with 1 being acceptable if not maintained or repeated for long periods) [10]. In this study, RULA was applied for each of the 18 informal workers, while performing each of the identified tasks, and the obtained RALs of the workers were calculated to obtain an average RAL per task, prior to extending the tool.

(ii) An extended RULA method

To provide a more detailed assessment of the workers, exposure value (EV) was determined for each task using duration and vibration (duration and vibration are not assessed by RULA).

A method of categorizing duration was developed from the information collected during the study (duration was defined as time spent in each task over a working day). Some of the tasks were conducted for 30 min, while other tasks were conducted for ±10 h (i.e., removing defects). In this study, the first step was to develop a system for ranking duration over a working day. Duration was ranked according to lowest to the highest exposure period (1: <1 h, 2: 1–3 h; 3: 3–7 h and 4: 7+ h) over a working day. In the current study, actions were repeated for around 12 times per minute or more. However, this was already accounted for by increasing the RULA score by 1 (actions repeated more than 4 times per minute can increase the RULA score (score = 1)). To obtain the EV, we multiplied the RAL by the duration of exposure, plus vibration (coded on a binary scale) adapted from a prior study [21]. The following equation was used:

EV = D × RAL +V(1)

where EV is the exposure value, RAL is the RULA action level per task (1 to 4, with 1 being the least exposed), D is the duration of exposure for each of the assessed activities (1: <1 h, 2: 1–3 h; 3: 3–7 h and 4: 7+ h) and V is the vibration (binary classification of present = 1/absent = 0).

**Figure 2:**
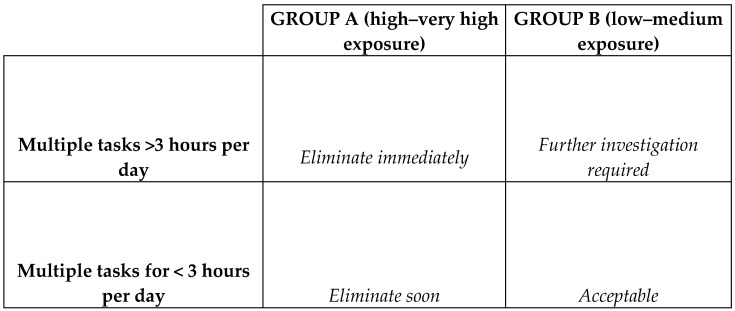
Action priority matrix.

The obtained EVs were used to determine exposure classification (EC) (1–2 (low), 3–6 (medium), 8–12 (high) or 16+ (very high)). The latter classifications were grouped according to high to very high exposure (Group A), and low to medium exposure (Group B). An Action priority matrix (Figure 2) was developed to guide intervention, and had four levels of assessing exposure: (i) high to very high exposure with multiple tasks conducted for >3 h per working day (action: eliminate immediately); (ii) high to very high exposure with multiple tasks conducted for <3 h per working day (action: eliminate soon); (iii) Low to medium exposure with multiple tasks conducted for >3 h per working day (action: further investigation required); and (iv) low to medium exposure with multiple tasks conducted for <3 h per day (action: acceptable). An action priority matrix is one of the widely used tools, and has been used in public health as a mechanism to prioritise public health problems [32,33].

## 3. Results

### 3.1. Demographic and Work Characteristics

All workers (*n* = 18) were male and between the ages of 19 and 61 years, mean (±SD) 36 (14) (Table 1). The majority (78%; *n* = 14) of the workers had never completed high school. Almost 40% (*n* = 7) of the workers had worked as cookware makers for a period ≥5 years. Some of the tasks were conducted for 30 min (preparing sand), while other tasks were conducted for ±10 h (i.e., removing defects). Of the 18 workers assessed, only 11% (*n* = 2) wore personal protection clothing (i.e., safety boots when preparing sand or goggles when removing cookware defects with a sanding machine). Sand casting was performed indoors in a structure built with corrugated metal sheeting. The main form of ventilation was a gap between the wall and the roof. The size of the working area ranged from 35 to 127 m^3^. The workers used different types of equipment including a spade (±2 kg), shovel (±3 kg), straight bar (±10 kg) and a long hand handled tool with a cup-shaped bowl for transporting molten metal from the furnace to the mould cavity (±10 kg). The tasks also involved lifting very heavy objects (above 20 kg) such as a mould cavity (created with sand) and casting.

### 3.2. Hand-Made Cookware Operation Process

The process of making cookware involved preparing sand (Table 2 (a)) by loosening sand and breaking up lumps in the soils (using a spade or a shovel) and preparing a flat sand surface (Table 2 (b)) for placing the mould cavity. Loading of the sand (Table 2 (c)) onto the replica of the mould cavity took place soon after, followed by creating mould cavity (Table 2 (d)) by compacting sand, (this was achieved by using a shovel and a straight bar). A casting box was used to cover the mould cavity; the workers then packed sand onto the excess space of the casting box. As soon as preparation of the mould cavity was complete, the artisans dismantled the casting box and sprinkled ash (Table 2 (e)) onto the mould cavity. Liquifying scrap metal (Table 2 (f)) took place in the furnace outdoors (in an open fire), then transporting and pouring molten metal into the mould (Table 2 (g)) was achieved by the use of a long hand handled cup shaped bowl to transport molten metal from the furnace to the workstation, and pouring onto the mould cavity to make casting. The final stage of cookware making involved breaking the mould (Table 2 (h)) by watering the mould to remove excess metal, breaking the mould to remove the casting, removing excess sand from the casting, and removing defects (Table 2 (i)) by cooling the final product and removing excess metal from the casting.

### 3.3. Risk Assessment

Ergonomic risk factors related to musculoskeletal disorders included repetitive motions, extending arms, bending and twisting, long static body postures and vibrations. The descriptive statistics of the RULA scores for each task are shown in Table 3.

The EV calculated using RAL, duration and vibration ranged from 3 to 17 (medium to very high exposure) (Table 4). Preparing sand, loading sand, transporting and pouring molten metal into the mould, dismantling the mould cavity, sprinkling ash and breaking the mould were at the highest RAL; however, these activities were conducted for less than an hour each. After taking duration into account, the latter activities were at the medium EC (Table 4). Preparing the flat sand surface, creating mould cavity and removing defects required investigation and changes soon, after taking account of the duration, and vibration (for removing defects only), the EC ranged from high to very high.

### 3.4. Action Priority Matrix

The action priority matrix was used to determine overall exposure and the actions to be taken (Figure 2). The workers performed all the tasks over a working day, and therefore, fell within the high to very high exposure domain of the action priority matrix, thus requiring immediate work changes.

### 3.5. Application of the Extended Tool in Other Informal Work Settings

**Figure 3:**
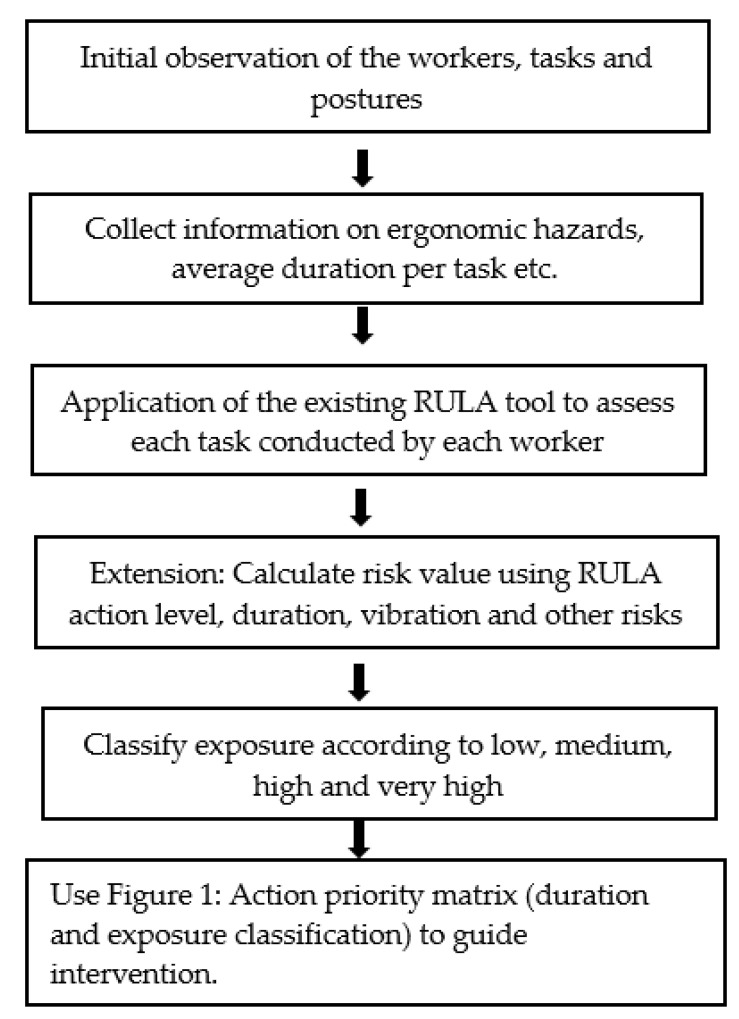
Details the application of the research in other informal work settings.

The methods applied in this research can be adopted and applied in other informal work settings using the process described in Figure 3 below.

## 4. Discussion

In this study, we were able to extend the RULA screening tool to provide a more detailed assessment of informal workers involved in hand-made cookware operations. According to the RALs, the hand-made cookware makers required: (i) investigation and changes soon; and (ii) immediate investigation and changes. The RALs observed from this informal sector craft are supported by previous studies conducted in formal workplace settings with similar physical exertions [1,2,7,34,35,36]. Extending the tool to include duration and vibration provided a range in EC of medium to very high exposure. The use of an action priority matrix to guide intervention was appropriate, and indicated that all the workers assessed were within the high to very high exposure domain.

The work conducted in the informal sector is highly variable within and between days [21,27,37]. The available conventional tools such as RULA [10], REBA [9], OWAS [14] and QEC [7], provide meaningful estimates, however, may be limited in assessing exposure for informal workers. Similarly, the use of the RULA screening tool to characterise exposure was meaningful in the current study, yet the tool could not sufficiently provide exposure estimates because of unstructured daily routines observed among the hand-made cookware makers. RULA has been reported to be useful in a variety of hand and machine packing operations [9]. The tool was based on the OWAS system (OWAS focuses on back, legs, feet, shoulder, arm and weight). After defining the work performed by the hand-made cookware makers, the use of RULA was appropriate. We addressed the RULA limitation by including duration, vibration; we also used the action priority matrix to determine overall exposure, and to guide the actions to be taken.

To address the limitation of the conventional risk assessment tools, Acquah et al. [37] developed an ergonomic assessment tool for informal e-waste collectors. The tool covered neck, trunk, upper limbs, lower limbs, force and repetition [37], and was based on the conventional ergonomic assessment tools [7,10,14,38]. Nevertheless, addressing the variable work schedules was not clearly stated in this study [37]. In the current study, the integration of the action priority matrix to guide intervention warrants adequate evaluation of informal workers with varying work schedules and tasks within days.

There is a dearth of information on ergonomic assessments among hand-made cookware makers and similar cottage industries. Studies conducted in the formal sector with similar physical exertion have reported similar outputs. For example, studies conducted in the United States and Brazil reported high to very high risks, and moderate to high risks among firefighters and emergency medical technicians [36], and textile industry workers [39], respectively. Physical exertions reported in these studies included working in the same position for long periods (standing, bent over, sitting, kneeling), working in awkward or cramped positions or working very fast for short periods (lifting, grasping, pulling, etc.) [36,39]. Similarly, in our study hand-made cookware makers worked more often in flexed postures than in upright postures. The tasks involved repetitive movements (around 12 times per minute or more), vibration (when removing cookware defects), forceful exertion, lifting, pulling and pushing.

The calculated EV resulted in a medium to very high EC. However, all the workers assessed in this study performed multiple tasks for >3 h per working day. Therefore, according to the action priority matrix, all the workers fall within the high to very high exposure domain and thus required immediate elimination of the hazards to control ergonomic exposure related to the potential for WRMDs. The hand-made cookware operation depends on product demand; thus, it is important to note that though the workers were within the high to very high exposure domain during the period of the study, it is likely that they are not exposed on a daily basis.

Workplaces with defined policies and control measures reduce WRMDs by engineering controls (i.e., workplace design) through the use of mechanical assist devices to relieve heavy load lifting and for changing the workstation layout (i.e., using height adjustable workbenches). In the informal sector, administrative controls are one of the feasible ways of controlling ergonomic risk factors; these control may include worker awareness programs on adjusting work schedules (i.e., taking short breaks during workdays), adjusting workloads, stretching the muscles before and during work, job rotation and tailor-made training programs on safe lifting techniques.

Physical exertion differed across the workers for each of the tasks assessed. For example, workers who were involved in trimming the cookware to remove defects either performed the task in seated positions or kneeled with one or both knees and the back was either flexed or side was bent. The differences in working postures may be explained by the lack of ergonomic risk awareness among the informal workers. In addition, though the workers could work in upright positions or seated positions in some instances, improper design and inappropriateness of the work station predominantly forced the workers to work in flexed postures, which led to an imbalanced and more kyphotic posture of the lumbar spine.

Vibration is believed to be an important contributor to WRMDs; for example, epidemiological studies have reported a significant relationship between whole-body vibration and low back pain [40,41]. In this study, only one task involved vibration, overall; however, all the workers were involved in removing cookware defects, and thus were exposed to vibrations.

The most important limitation of the present study was the inability to collect data related to musculoskeletal disorders; however, this study presents a way forward in terms of research required. It currently remains unclear which musculoskeletal disorders are frequent among hand-made cookware makers. In addition, though the workers were at the highest exposure domain, there is a variation in the length of time an individual perceives discomfort; for example, workers may adjust their working posture to relieve loading; thus, there is an urgent need for carefully designed studies and interventions focusing on WRMDs, anthropometric data, habits and work experience etc. related to ergonomic hazards. Informal workers used a range of postures; because of this, the selection of posture was based on the postures sustained for the longest periods (i.e., squatting). Future research should focus on the most feasible way for analyzing work postures among informal workers. The risk assessment for this study indicate that immediate action is required; however, there are challenges in actually implementing the findings of this study, especially by the informal workers because of the lack of resources. Research translation related to ergonomic hazards, risks, exposure and ways to mitigate the latter may be an alternative.

Observational risk assessment was used to determine ergonomic risks, though this method is prone to significant measurement errors [42]. Improvements in ergonomic risk assessments include the introduction of an electrogoniometer and surface electromyography which measures postural levels [26,42]. These tools are able to accurately and reliably measure physical exposures in the workplace. However, the use of a workplace observational approach is a cost-effective method for evaluating exposure among informal workers.

## 5. Conclusions

In this study involving hand-made cookware makers, we were able to apply and extend an existing conventional risk assessment tool by including duration, vibration and an action priority matrix to determine overall exposure and actions to be taken. We assessed nine tasks conducted by the informal workers. The results showed that all the workers were at the highest exposure domain, and required immediate changes. RULA proved to be an effective and reliable ergonomic screening method for identifying ergonomic factors related to musculoskeletal disorders among hand-made cookware makers. Ergonomic risk awareness is probably the most effective means for reducing exposure among hand-made cookware makers. Our study included six hand-made cookware operation sites; therefore, future research should focus on the application of these methods in other similar informal sector crafts, with similar or different work profiles, and ergonomic hazards. Extending the RULA tool inevitably shines a light on the exposure of informal workers to ergonomic hazards by taking duration, vibrations and different work practices into consideration.

## Figures and Tables

**Table 1:** Demographic characteristics.

Variable	*n* (%)
Age: mean (SD)	36 (14)
Gender: Male	18 (100)
Education: never completed high school	14 (78)
Personal protective equipment	2 (11)
Working period: ≥5 years	7 (40)

**Table 2:** Description of the tasks and ergonomic hazards.

Description of the Activity			Description of the Hazard				
			Back	Arm	Legs	Wrist	Instrumentation/Load
Preparing sand	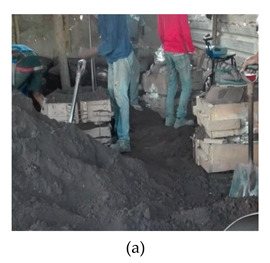	Loosening sand and breaking up lumps in the soils	The movement of the back is very frequent (around 12 times per minute or more)	Repetitive movements of the arms and lifting weights above shoulder height (12 times per minute or more)	Body weight is evenly distributed over both feet, with room for changes or position	Repetitive movements of the wrist (12 times per minute or more with 15° or more in either flexion or extension	Spade or shovel, maximum force exerted was ±2 kg
Preparing flat sand surface	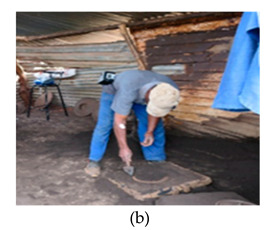	Preparing a flat sand surface to make a bed for the mould cavity	The range in movement of the trunk is 60° or more flexion (12 times per minute or more)	Repetitive movements of the arms (12 times per minute or more)	Body weight is evenly distributed over both feet, with room for changes or position	Repetitive movements of the wrist. The task involves 0–15° in either flexion or extension (12 times per minute or more)	The maximum force exerted was ±2 kg
Loading sand	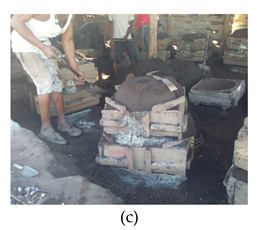	Loading sand onto the replica of the cookware and the casting box to create mould cavity	The range in movement of the trunk is at or between 20–60° flexion. In some instances, the back is excessively flexed or twisted or the side is bent. The movement of the back is very frequent (around 12 times per minute or more)	Repetitive movements of the arms (12 times per minute or more)	Body weight is evenly distributed over both feet, with room for changes or position	Repetitive movements of the wrist. The task involves 0–15° in either flexion or extension (12 times per minute or more)	Spade, maximum weight of between 3 and 5 kg
Creating mould cavity	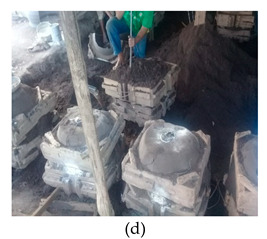	Packing sand onto the replica of the cookware to make a mould cavity and ramming sand onto the excess space of the casting box	The range in movement of the trunk is at or between 20–60° flexion. In some instances, the back is excessively flexed or twisted or side bent. The movement of the back is very frequent (around 12 times per minute or more)	Lifting weights above shoulder height and repeating similar motion patterns more than 12 times per minute	Kneeling on one or both knees	Repetitive movements of the wrist. The task involves 0–15° in either flexion or extension (12 times per minute or more)	Shovel and a 7-kg rod is used for ramming the sand
Dismantle the casting box and sprinkling ash	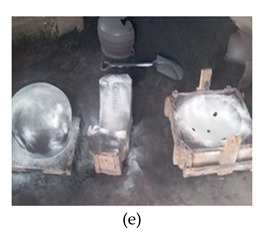	Dismantling the casting box and sprinkling ash onto the mould cavity and flat sand surface	Moderately/excessively flexed or twisted or side bent	Lower arm working across the midline of the body or out to the side; in most cases, the hands are at or below waist height	Standing or squatting with one knee bent or kneeling on one or more knees	The task involves a deviated or bent wrist	The maximum weight handled manually ranges from light (5 kg or less), moderate (6 to 10 kg), heavy (11 to 20 kg) and very heavy (more than 20 kg)
Liquefying scrap metal	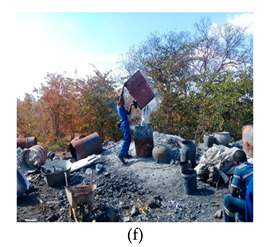	Liquifying scrap metal (i.e., used car and motor bike engine parts, waste aluminium and computer components)	Moderately flexed	Repetitive movements of the arms (12 times per minute or more)	Body weight is evenly distributed over both feet, with room for changes or position.	Repetitive movements of the wrist. The task involves 0–15° in either flexion or extension (12 times per minute or more)	Handling maximum weight of ±10 kg
Transporting and pouring molten metal into the mould	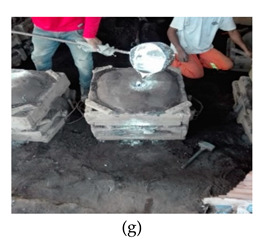	Transporting molten metal from the furnace to the workstation and pouring molten metal into the mould cavity	The back remains in a static position during the pouring of molten metal	Static posture of the arms	Body weight is evenly distributed over both feet, with room for changes or position	Static posture of the wrist	Long hand handled cup shaped bowl maximum weight of between 11 to 20 kg. The load of more than 10 kg is static for more than 1 min
Breaking the mould	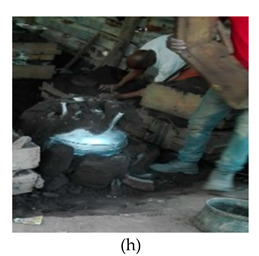	Breaking the mould	The range in movement of the trunk is at or between 20–60° flexion. In some instances, the back is excessively flexed or twisted or side bent. The movement of the back is very frequent (around 12 times per minute or more)	Repetitive movements of the arms (more than 12 times per minute)	Body weight is evenly distributed over both feet, with room for changes or positions	Repetitive movements of the wrist (around 12 times per minute or more)	Spade or 7 kg rod, maximum weight of between 3 and 5 kg
Removing defects	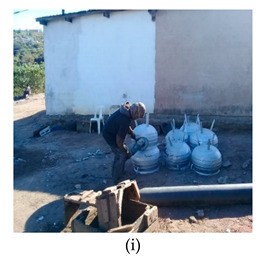	Inspection of the final product to remove any defects by removing sand, excess metal from the casting	Moderately/excessively flexed or twisted or side bent, vibration	Repetitive movements of the arms, (around 12 times per minute or more) and vibration	Body weight is evenly distributed over both feet, with room for changes or position, vibration	Repetitive movements of the wrist (around 12 times per minute or more) and vibration	Sanding machine, maximum weight of more than 20 kg.

**Table 3:** RULA action levels for each task.

Task	Mean (SD)	Median	Minimum	Maximum
Preparing sand	4 (0.0)	4	-	4
Preparing flat sand surface	4 (0.6)	4	2	4
Loading sand	4 (0.3)	4	3	4
Creating mould cavity	4 (0.3)	4	3	4
Dismantling the mould cavity and sprinkling ash	3 (0.3)	3	2	3
Liquifying metal	3 (0.5)	3	2	3
Transporting and pouring molten metal into the mould	3 (0.9)	3	2	4
Breaking the mould	3 (0.8)	3	2	4
Removing defects	4 (0.0)	4	-	4

**Table 4:** Ergonomic assessment.

Task	* Average RULA Action Level	** Duration Per Task	*** Vibration	^#^ Exposure Value	^##^ Exposure Classification
Preparing sand	4	1	0	4	Medium
2. Preparing flat sand surface	4	2	0	8	High
3. Loading sand	4	1	0	4	Medium
4. Creating mould cavity	4	3	0	12	High
5. Dismantling the mould cavity and sprinkling ash	3	1	0	3	Medium
6. Liquifying metal	3	2	0	3	Medium
7. Transporting and pouring molten metal into the mould	3	1	0	3	Medium
8. Breaking the mould	3	1	0	3	Medium
9. Removing defects	4	4	1	17	Very high

* The RULA scores were calculated by assessing each of the 18 workers while conducting each task, and providing average RULA scores in relation to each task. 4 = A score of 7 indicates that investigation and changes are required immediately, 3 = A score of 5 or 6 indicates that investigation and changes are required soon; 2 = A score of 3 or 4 indicates that further investigation is needed and changes may be required; 1 = A score of 1 or 2 indicate that posture is acceptable if it is not maintained or repeated for long period. ** average duration per task: 4 = 7+ h; 3 = 3–7 h; 2 = 1–3 h; 1 = <1 h. *** vibration: 0 = absent, 1 = present; ^#^ Exposure value: RULA Action level × duration per task + vibration. ^##^ Exposure classification: 16+ = very high exposure; 8–12 = high exposure; 3–6 = medium exposure; 1–2 = low exposure.

## Data Availability

The data presented in this study are available on request from the corresponding author. The data are not publicly available due to ethical issues.

## References

[1] Choobineh A., Tabatabaei S.H., Mokhtarzadeh A., Salehi M. (2007). Musculoskeletal problems among workers of an Iranian rubber factory. J. Occup. Health.

[2] Punnett L., Wegman D.H. (2004). Work-related musculoskeletal disorders: The epidemiologic evidence and the debate. J. Electromyogr. Kinesiol..

[3] Sim J., Lacey R.J., Lewis M. (2006). The impact of workplace risk factors on the occurrence of neck and upper limb pain: A general population study. BMC Public Health.

[4] Bernard B. A Critical Review of Epidemiologic Evidence for Work-Related Musculoskeletal Disorders of the Neck, Upper Extremity, and Low Back. https://www.cdc.gov/niosh/docs/97-141/.

[5] Vos T., Flaxman A.D., Naghavi M., Lozano R., Michaud C., Ezzati M., Shibuya K., Salomon J.A., Abdalla S., Aboyans V. (2012). Years lived with disability (YLDs) for 1160 sequelae of 289 diseases and injuries 1990–2010: A systematic analysis for the Global Burden of Disease Study 2010. Lancet.

[6] Hoy D., March L., Brooks P., Blyth F., Woolf A., Bain C., Williams G., Smith E., Vos T., Barendregt J. (2014). The global burden of low back pain: Estimates from the Global Burden of Disease 2010 study. Ann. Rheum. Dis..

[7] David G., Woods V., Li G., Buckle P. (2008). The development of the Quick Exposure Check (QEC) for assessing exposure to risk factors for work-related musculoskeletal disorders. Appl. Ergon..

[8] MacDonald L., Karasek R., Punnett L., Scharf T. (2001). Covariation between workplace physical and psychosocial stressors: Evidence and implications for occupational health research and prevention. Ergonomics.

[9] McAtamney L., Hignett S. REBA: A rapid entire body assessment method for investigating work related musculoskeletal disorders. Proceedings of the 31st Annual Conference of the Ergonomics Society of Australia.

[10] McAtamney L., Corlett E.N. (1993). RULA: A survey method for the investigation of work-related upper limb disorders. Appl. Ergon..

[11] Steven Moore J., Garg A. (1995). The strain index: A proposed method to analyze jobs for risk of distal upper extremity disorders. Am. Ind. Hyg. Assoc. J..

[12] Waters T.R., Putz-Anderson V., Garg A., Fine L.J. (1993). Revised NIOSH equation for the design and evaluation of manual lifting tasks. Ergonomics.

[13] Karhu O., Kansi P., Kuorinka I. (1977). Correcting working postures in industry: A practical method for analysis. Appl. Ergon..

[14] Sesek R., Gilkey D., Rosecrance J., Guzy A. The Utility of OWAS in Auto Manufacturing Assembly Job Evaluations. https://www.academia.edu/5314247/the_utility_of_owas_in_auto_manufacturing_assembly.

[15] Roman-Liu D., Groborz A., Tokarski T. (2013). Comparison of risk assessment procedures used in OCRA and ULRA methods. Ergonomics.

[16] Roman-Liu D. (2005). Upper limb load as a function of repetitive task parameters: Part 1—A model of upper limb load. Int. J. Occup. Saf. Ergon..

[17] Roman-Liu D., Tokarski T. (2005). Upper limb load as a function of repetitive task parameters: Part 2—An experimental study. Int. J. Occup. Saf. Ergon..

[18] Chander D.S., Cavatorta M.P. (2017). An observational method for postural ergonomic risk assessment (PERA). Int. J. Ind. Ergon..

[19] Tangkittipaporn J., Jiangsathaporn W. (2017). Musculoskeletal pain and mental agony reacting to ergonomic risks in the Thai informal working environment. Psychology.

[20] De Sousa M.N.A., de Oliveira Santos B.M., Zaia J.E., Bertoncello D., Feitosa A.D.N.A., de Assis E.V., Batista H.M.T., de Mello Monteiro C.B., Maia P.C.G.G.S., Bezerra I.M.P. (2015). Musculoskeletal disorders in informal mining workers. Int. Arch. Med..

[21] Acquah A., Arko-Mensah J., D’Souza C., Martin B., Quakyi I., Robins T., Fobil J. (2019). Prevalence of work-related musculoskeletal disorders among e-waste workers at Agbogbloshie in Accra, Ghana. Environ. Epidemiol..

[22] ILO Safety and Health at Work: A Vision for Sustainable Prevention. In Proceedings of the XX World Congress on Safety and Health at Work 2014, Frankfurt, Germany, 24–27 August 2014. https://www.ilo.org/global/topics/safety-and-health-at-work/resources-library/publications/WCMS_301214/lang--en/index.htm.

[23] Forastieri V. Improvement of Working Conditions and Environment in the Informal Sector through Safety and Health Measures. https://www.ilo.org/safework/info/publications/WCMS_110306/lang--en/index.htm.

[24] WHO (1994). Global Strategy on Occupational Health for All: The Way to Health at Work, Recommendation of the Second Meeting of the WHO Collaborating Centres in Occupational Health, Beijing, China, 11–14 October 1994.

[25] Takala E.-P., Pehkonen I., Forsman M., Hansson G.-Å., Mathiassen S.E., Neumann W.P., Sjøgaard G., Veiersted K.B., Westgaard R.H., Winkel J. (2010). Systematic evaluation of observational methods assessing biomechanical exposures at work. Scand. J. Work. Environ. Health.

[26] Wang W.-X., Sun S.-Q., Tang Z.-C. (2018). Comparison of contact interface factors for surface electromyography control wearable device. Adv. Mech. Eng..

[27] Shezi B., Naidoo R.N., Muttoo S., Mathee A., Alfers L., Dobson R., Ndlovu P., Street R.A. (2019). Informal-sector occupational hazards: An observational workplace assessment of the traditional medicine trade in South Africa. Int. J. Occup. Saf. Ergon..

[28] Guma T.N., Uche L.O. (2019). Sand Mould Design for Casting an Aluminium Pot-A Basic Procedure of Supplementing Artisanal Practices. Eur. J. Eng. Res. Sci..

[29] Guma T., Uche O.L. (2019). A typification of foundry practices for correct artisanal sand casting of aluminum pots. Engineering.

[30] Street R.A., Mathee A., Tanda S., Hauzenberger C., Naidoo S., Goessler W. (2020). Recycling of scrap metal into artisanal cookware in the informal sector: A public health threat from multi metal exposure in South Africa. Sci. Total Environ..

[31] Shezi B., Mathee A., Cele N., Ndabandaba S., Street R.A. (2020). Occupational Exposure to Fine Particulate Matter (PM4 and PM2. 5) during Hand-Made Cookware Operation: Personal, Indoor and Outdoor Levels. Int. J. Environ. Res. Public Health.

[32] Park S., Vega R., Choto Z., Grewe M. (2010). Risk-based asset prioritization of water transmission/distribution pipes for the City of Tampa. Fla. Water Resour. J..

[33] Ramani V.K. (2021). Prioritization matrix for the diabetes prevention and control program: A concept paper. J. Diabetol..

[34] Wahlström J. (2005). Ergonomics, musculoskeletal disorders and computer work. Occup. Med..

[35] Thiehoff R. (2002). Economic significance of work disability caused by musculoskeletal disorders. Der Orthop..

[36] Gentzler M., Stader S. (2010). Posture stress on firefighters and emergency medical technicians (EMTs) associated with repetitive reaching, bending, lifting, and pulling tasks. Work.

[37] Acquah A.A., D’Souza C., Martin B., Arko-Mensah J., Asabea Nti A., Kwarteng L., Takyi S., Botwe P.K., Tettey P., Dwomoh D. Development of an Observation-Based Tool for Ergonomic Exposure Assessment in Informal Electronic Waste Recycling and Other Unregulated Non-Repetitive Work. https://www.researchgate.net/publication/349144912_.

[38] Middlesworth M. Rapid Entire Body Assessment (REBA). https://ergo-plus.com/reba-assessment-tool-guide/.

[39] Comper M.L.C., Padula R.S. (2013). Ergonomic risk assessment among textile industry workers using two instruments: Quick Exposure Check and Job Factors Questionnaire. Educação Pesquisa.

[40] Lind C. Assessment and Design of Industrial Manual Handling to Reduce Physical Ergonomics Hazards: Use and Development of Assessment Tools. https://www.diva-portal.org/smash/get/diva2:1094542/FULLTEXT02.

[41] Bovenzi M., Hulshof C. (1999). An updated review of epidemiologic studies on the relationship between exposure to whole-body vibration and low back pain (1986–1997). Int. Arch. Occup. Environ. Health.

[42] Lowe B.D. (2004). Accuracy and validity of observational estimates of wrist and forearm posture. Ergonomics.

